# Supplementary material for: Chromophore charge-state switching through copper-dependent homodimerisation of an engineered green fluorescent protein
Source: Chem Sci. 2025 Oct 13;16(46):22136–46. doi: 10.1039/d5sc06589e (PMC12547689; doi:10.1039/d5sc06589e)
Supplement: SC-016-D5SC06589E-s001 [file SC-016-D5SC06589E-s001.pdf]

Supplementary Information (SI) for Chemical Science.
This journal is © The Royal Society of Chemistry 2025

Chromophore charge-state switching through copper-dependent homodimerisation of an engineered green fluorescent protein.

Rochelle D Ahmed[a#], Danoo Vitsupakorn[a#], Kieran D. Hartwell[a], Karma Albalawi[a,c], Pierre Rizkallah[b], Peter D. Watson[a] and D. Dafydd Jones[a*].

a. School of Biosciences, Molecular Biosciences Division, Cardiff University, Sir Martin Evans Building, Cardiff, CF10 3AX, UK.
b. School of Medicine, Cardiff University, Cardiff, CF14 4XN, UK.
c. Department of Chemistry, Faculty of Science, University of Tabuk, Tabuk, Saudi Arabia.

Supplementary Information.

Supporting Methods

Sequence of the GFP-diS2-ZIP resident in pET28a.

ATGGTTAGCAAAGGTGAAGAACTGTTTACCGGCGTTGTGCCGATTCTGGTGGA
ACTGGATGGTGATGTGAATGGCCATAAATTTAGCGTTCGTGGCGAAGGCGAAG
GTGATGCGACCAACGGTAAACTGACCCTGAAATTTATTTGCACCACCGGTAAAC
TGCCGGTTCCGTGGCCGACCCTGGTGACCACCCTGACCTATGGCGTTCAGTGC
TTTAGCCGCTATCCGGATCATATGAAACGCCATGATTTCTTTAAAAGCGCGATG
CCGGAAGGCTATGTGCAGGAACGTACCATTAGCTTCAAAGATGATGGCACCTAT
AAAACCCGTGCGGAAGTTAAATTTGAAGGCGATACCCTGGTGAACCGCATTGAA
CTGAAAGGTATTGATTTTAAAGAAGATGGCAACATTCTGGGTCATAAACTGGAAT
ATAATTTCAACAGCTGCAATGTGTATATTACCGCCGATAAACAGAAAAATGGCAT
CAAAGCGAACTTTAAAATCCGTCACAACGTGGAAGATGGTAGCGTGCAGCTGG
CGGATCATTATCAGCAGAATACCCCGATTGGTGATGGCCCGGTGCTGCTGCCG
GATAATCATTATCTGAGCACCCAGAGCTTTCTGAGCAAAGATCCGAATGAAAAA
CGTGATCATATGGTGCTGCTGGAATTTGTTACCGCCGCGGGCATTACCCACGG
TATGGATGAACTGTATAAAGGCAGCACCAAAGATAAAGAAAACCTGTATTTTCA
GTCTAAGCAGCTTGAGGACAAAGTCGAGGAACTGCTATCCAAGAACTATCATCT
GGAGAACGAGGTGGCTCGTCTGAAGAAACTGGTGGGTGGTTCTCACCATCATC
ATCACCATTAA

Protein purification.

The wild-type sfGFP and it’s variants in the pBAD vector (Amp^R) were transformed into *E. coli* Top 10^{TM} (Invitrogen, Paisley, UK). The pET28a GFP-diS2-ZIP (Kan^R) and the pCA24N P50-GFP (Cm^R) variants were transformed into *E. coli* BL21 (DE3) (NE Biolabs) for recombinant protein expression. A single colony was taken from the transformation and used to inoculate a 10 mL 2xYT starter culture supplemented with suitabble selective antibiotic. The starter cultures were placed in a shaking incubator overnight at 37 °C. For the pBAD-based variants, the starter culture was used to inoculate 1L culture of autoinduction media supplemented with ampicillin (50

μg/mL) [1]. The autoinduction cultures were incubated for a further 18-24 hr at 37°C. For the pCA24N P50-GFP variants, 1L of 2xYT supplemented with chloramphenicol (35 μg/mL) was inoculated with the starter culture and incubated at 37 °C with shaking (200 RPM). At an OD_{600} of ~0.8, IPTG was added to a final concentration of 0.4 mM and incubated overnight with shaking at 28 °C. The cells were then pelleted by centrifugation and then resuspended 50 mM Tris-HCl, pH 8.0. Cells were lysed using the French Pressure Cell. Soluble cell lysate was then separated from insoluble fractions via centrifugation at 25,000*g* for 40 mins. Clarified cell lysate was passed through a 5 mL His Trap™ HP column (Cytiva) equilibrated in 50 mM Tris-HCl, pH 8.0 buffer containing 10 mM imidazole. Bound target protein was then eluted by the addition of the 50 mM Tris-HCl, pH 8.0 buffer containing imidazole at a gradient from 10 to 500 mM. Samples of each fraction were taken and used to check for purity via SDS-PAGE. When required size exclusion chromatography (SEC) was performed to further purify samples and to analyse the oligomeric state. SEC was performed with either HiLoad™ 16/600 Superdex™ S75 pg (Cytiva) or HiLoad™ 16/600 Superdex™ S200 pg (Cytiva).

Table S1. Crystallographic statistics from x-ray diffraction refinement and final bond parameters for the GFP-diS2 crystal structures

	**mGFP-diS2**	**dGFP-diS2**
	**Data collection/reduction statistics**	
PDB ID	8c1x	8bxp
Wavelength (Å)	0.81532	0.81532
Crystallisation	Vapour Diffusion, Sitting Drop, 0.2 M Na malonate dibasic monohydrate, 0.1 M Bis-Tris propane, pH 7.5, 20%w/v PEG 3350	Vapour Diffusion, Sitting Drop, 0.2 M sodium acetate trihydrate, 0.1 M Bis-tris propane, pH 7.5, 20%w/v PEG 3350
Beamline (DLS, UK)	I03	I03
Space group	$P\ 1\ 2_1\ 1$	$P\ 2\ 2\ 2_1$
a (Å)	67.49	67.8
b (Å)	73.76	75.02
c (Å)	127.28	125.78
Resolution range (Å)	39.25 - 1.89	50.31 - 1.79
Total reflections measured	707,104	826,980
Unique reflections	100,181	61,413
Completeness (%) (last shell)	100 (100)	99.8 (95.7)
I/σ (last shell)	10.4 (0.3)	10.5 (0.4)
R(merge)(%) (last shell)	9.3 (329.2)	0.125 (4.902)
B (iso) from Wilson (Å2)	35.3	27.68
	**Refinement statistics**	
R-factor[b] (%)	20.6	20.0
R-factor[c] (%)	24.7	23.4
RMSD bond lengths (Å)	0.0121	0.013
RMSD bond angles (°)	1.806	1.795
	**Ramachandran Plot statistics**	
Favored region (%)	93	96.68
Allowed region (%)	6.4	2.65
Disallowed region (%)	0.6	0.66

Table S2. Residues comprising the dGFP-diS2 dimer interface as determined using PISA [2]

<table>
<tr><th colspan="3">Chain A</th><th colspan="3">Chain B</th></tr>
<tr><th>Residue</th><th>H-bond</th><th>BSA (Å²)[a]</th><th>Residue</th><th>H-bonds</th><th>BSA (Å²)[a]</th></tr>
<tr><td>T38</td><td></td><td>0.99 |</td><td>T38</td><td></td><td>1.11 |</td></tr>
<tr><td>N39</td><td>H</td><td>33.64 |||||</td><td>N39</td><td>H</td><td>33.11 |||||</td></tr>
<tr><td>R73</td><td>H</td><td>90.31 |||||||||</td><td>R73</td><td>H</td><td>90.49 |||||||||</td></tr>
<tr><td>P75</td><td></td><td>35.66 |||||||||</td><td>P75</td><td></td><td>35.66 |||||||||</td></tr>
<tr><td>H77</td><td></td><td>23.80 |||||</td><td>H77</td><td></td><td>24.25 |||||</td></tr>
<tr><td>M78</td><td></td><td>0.83 ||||||||||</td><td>M78</td><td></td><td>0.83 ||||||||||</td></tr>
<tr><td>N146</td><td>H</td><td>9.04 ||</td><td>N146</td><td>H</td><td>9.17 ||</td></tr>
<tr><td>S147</td><td></td><td>3.48 ||</td><td>S147</td><td></td><td>3.36 ||</td></tr>
<tr><td>C148</td><td></td><td>55.15 |||||</td><td>C148</td><td></td><td>55.48 |||||</td></tr>
<tr><td>N149</td><td></td><td>20.93 |||||</td><td>N149</td><td></td><td>20.54 |||||</td></tr>
<tr><td>Y200</td><td>H</td><td>27.14 ||||||</td><td>Y200</td><td>H</td><td>27.09 ||||||</td></tr>
<tr><td>S202</td><td>H</td><td>24.80 ||||||||</td><td>S202</td><td>H</td><td>24.93 ||||||||</td></tr>
<tr><td>Q204</td><td>H</td><td>90.68 |||||||||</td><td>Q204</td><td>H</td><td>90.41 |||||||||</td></tr>
<tr><td>S205</td><td></td><td>5.11 |||</td><td>S205</td><td></td><td>5.20 |||</td></tr>
<tr><td>F206</td><td></td><td>107.03 ||||||||||</td><td>F206</td><td></td><td>107.32 ||||||||||</td></tr>
<tr><td>L207</td><td></td><td>16.74 ||||||||</td><td>L207</td><td></td><td>17.00 ||||||||</td></tr>
<tr><td>S208</td><td></td><td>37.49 |||||||</td><td>S208</td><td></td><td>37.31 |||||||</td></tr>
<tr><td>K209</td><td></td><td>3.69 |</td><td>K209</td><td></td><td>3.81 |</td></tr>
<tr><td>V219</td><td></td><td>7.83 ||</td><td>V219</td><td></td><td>8.00 ||</td></tr>
<tr><td>L221</td><td></td><td>34.80 |||||||</td><td>L221</td><td></td><td>34.97 |||||||</td></tr>
<tr><td>F223</td><td></td><td>68.67 |||||||||</td><td>F223</td><td></td><td>67.88 |||||||||</td></tr>
<tr><td>V224</td><td></td><td>0.68 |</td><td>V224</td><td></td><td>0.68 |</td></tr>
<tr><td>T225</td><td></td><td>29.93 ||||||||</td><td>T225</td><td></td><td>30.09 ||||||||</td></tr>
<tr><td>A226</td><td></td><td>0.48 ||||||||||</td><td>A226</td><td></td><td>0.36 ||||||||</td></tr>
<tr><td>A227</td><td></td><td>33.71 ||||||||||</td><td>A227</td><td></td><td>33.88 ||||||||||</td></tr>
<tr><td>G228</td><td></td><td>4.91 |</td><td>G228</td><td></td><td>4.46 |</td></tr>
<tr><td>I229</td><td></td><td>3.54 ||</td><td>I229</td><td></td><td>3.78 ||</td></tr>
<tr><td>T230</td><td></td><td>49.18 |||||</td><td>T230</td><td></td><td>48.90 |||||</td></tr>
<tr><td>M233</td><td></td><td>90.80 ||||||</td><td>M233</td><td></td><td>90.46 ||||||</td></tr>
</table>

[a], Buried surface area. Each vertical line represents percentage of buried surface area, with one bar equal to 10%.

Table S3. H-bonds at the dGFP-diS2 dimer interface as determined by PISA[2]

Chain A			Chain B	
**Residue**	**Atoms**[a]	**Distance (Å)**	**Atoms**[a]	**Residue**
N39	O	2.95	NH1	N73
R73	NH1	2.95	O	N39
N146	OD1	3.89	OH	Y200
Y200	OH	3.89	OD1	N146
Y200	OH	2.16	OE1	N204
S202	OG	3.37	OG	N202
Q204	OE1	2.16	OH	Y200

a, atoms are labelled by their PDB nomenclature.

Table S4. Change in emission ratio of GFPdiS2-ZIP in the presence of various metal ions

Metal	Ex 483 nm:Ex 388 nm		R/R_0
	Before[a]	After[b]	
$CaCl_2$	0.61	0.71	1.16
$MgCl_2$	0.65	0.69	1.05
$MnCl_2$	0.75	0.85	1.13
$ZnCl_2$	1.15	1.18	1.03
$ZnSO_4$	1.12	1.12	1.00
Fe^{3+}: $FeCl_3$[b]	2.04	2.49	1.22
Fe^{2+}: $FeSO_4$[b]	4.26	4.08	0.96
$CoCl_2$	0.66	0.77	1.16

a, the emission ratio immediately after (<30 sec) of 1mM metal ion.
b, 1 hr after addition.
c, higher ratio due to increase in emission on excitation at the higher wavelength due to the metal ion itself.

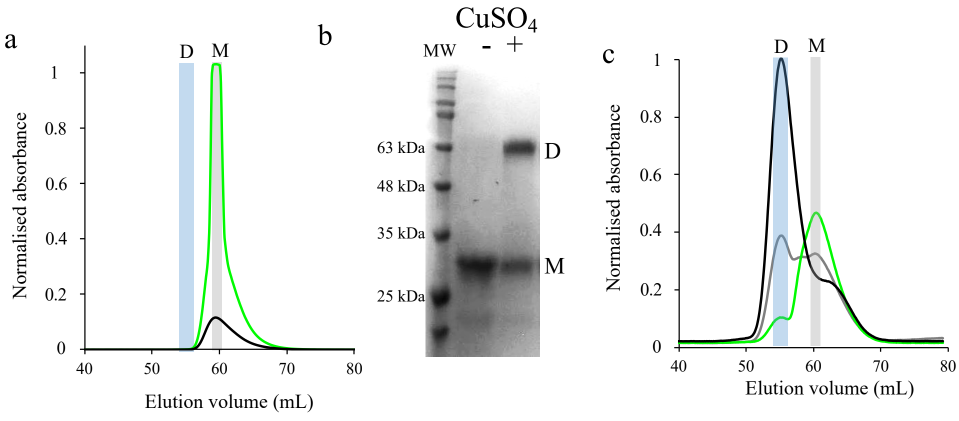

Fig. S1. Dimerisation characteristics of GFP-diS1. (a) Size exclusion chromatography (SEC) of purified GFP-diS1. (b) Non-reducing SDS-PAGE of GFP-

diS1 in the absence (-) and presence (+) 1 mM $CuSO_4$. The lower band present just below 25 kDa is commonly observed in fluorescent proteins due to cleavage in the chromophore on heat denaturation [3,4]. (c) Separation of dimer and monomer species by SEC after incubation with $CuSO_4$. On all figures, D signifies dimer and M monomer. Green, black and grey lines in the SEC elution profiles represent absorbance at 400 nm, 485nm and 280 nm, respectively.

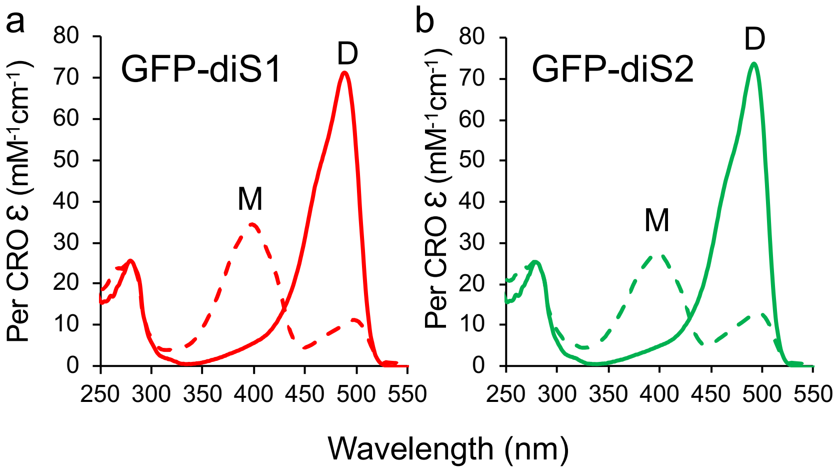

Fig. S2. Absorbance spectra of (a) GFP-diS1 and (b) GFP-diS2 in their monomer (dashed lines) or dimer (solid lines) forms. Absorbance is shown as the per chromophore molar absorbance coefficient for direct comparison.

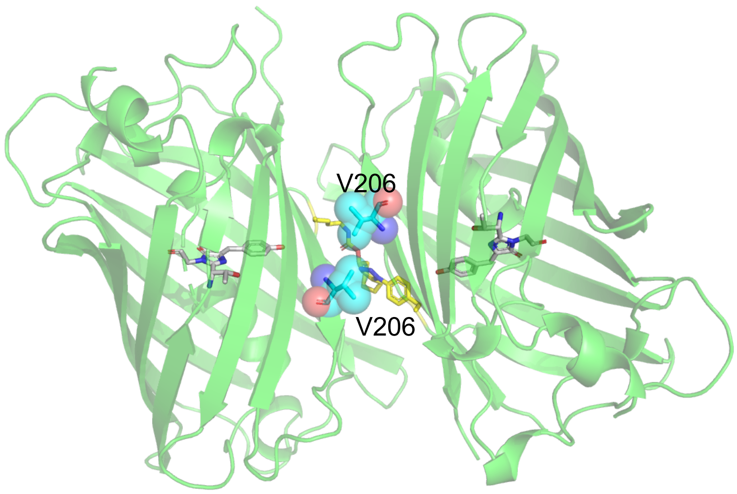

Fig. S3. The position of V206 at the dimer interface of a sfGFP homodimer linked by alkyne-azide crosslink at residue 148 (PDB: 5nhn) [5].

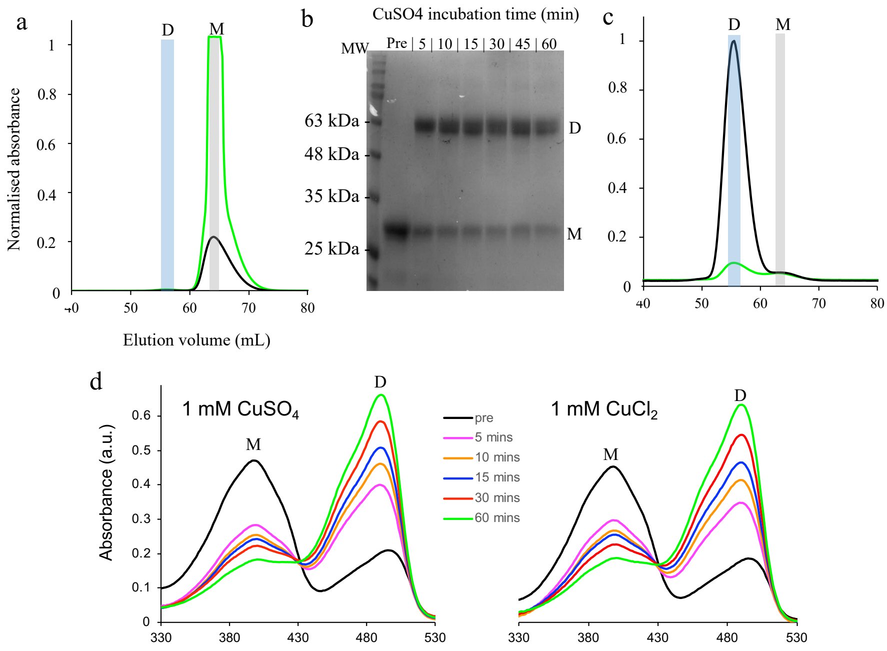

Fig. S4. Dimerisation characteristics of GFP-diS2. (a) Size exclusion chromatography (SEC) of purified GFP-diS2. Green and black lines in the SEC elution profiles represent absorbance at 400 nm and 485nm, respectively. (b) Non-reducing SDS-PAGE of GFP-diS2 on addition of 1 mM $CuSO_4$. Samples were taken periodically after $CuSO_4$ addition. "Pre" signifies prior to $CuSO_4$ addition. The lane numbers are time of incubation in minutes. (c) Separation of dimer and monomer species by SEC after incubation with $CuSO_4$. (d) Change in absorbance spectra on addition of either 1 mM $CuSO_4$ or 1 mM $CuCl_2$ to 10 μM GFP-diS2. On all figures, D signifies dimer and M monomer. SEC was performed with HiLoad™ 16/600 Superdex™ S200 pg (Cytiva).

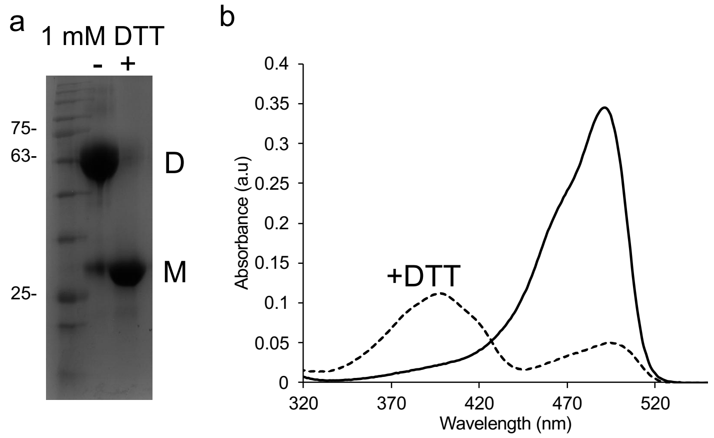

Fig. S5. The effect of DTT on dGFP-diS2. (a) Non-reducing SDS-PAGE of the dimer before (-) and after (+) incubation of dGFP-diS2 for 5 min in 1 mM DTT. (b) Corresponding absorbance spectra (5 μM protein) before (solid line) and after (dashed line) incubation for 5 min with 1 mM DTT.

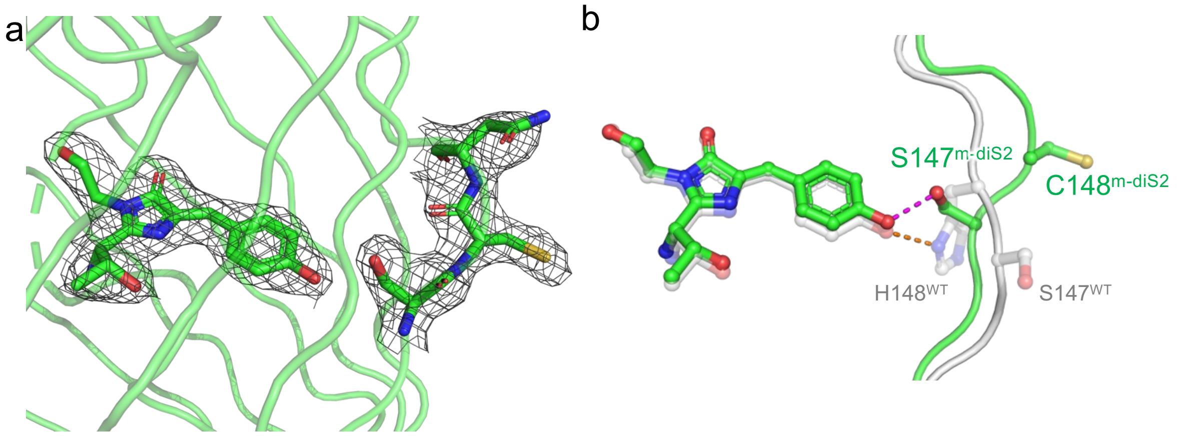

Fig. S6. (a) The $2F_0$-F_c map (σ 1.0) of the chromophore and residues S147 and C148 in mGFP-diS2. (b) Comparison of the chromophore and residues 147 and 148 in WT sfGFP (grey; PDB 2b3p [6]) and mGFP-diS2 (green).

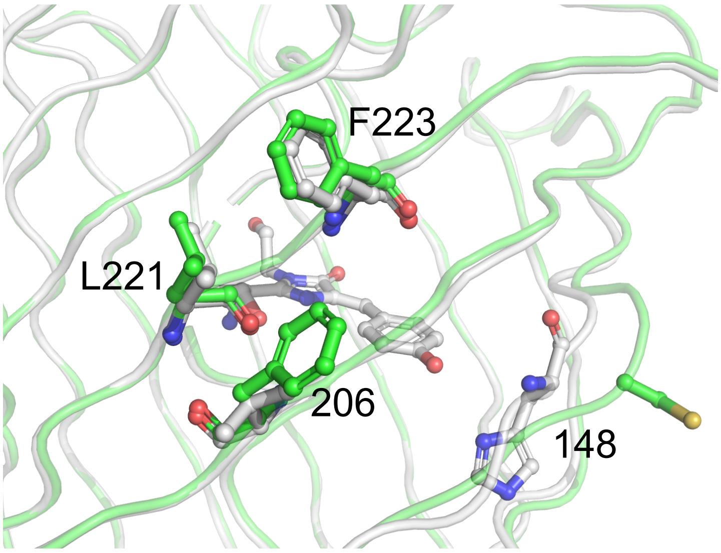

Fig. S7. Structural comparison of WT sfGFP (grey) and mGFP-diS2 (green) centred around the V206F mutation.

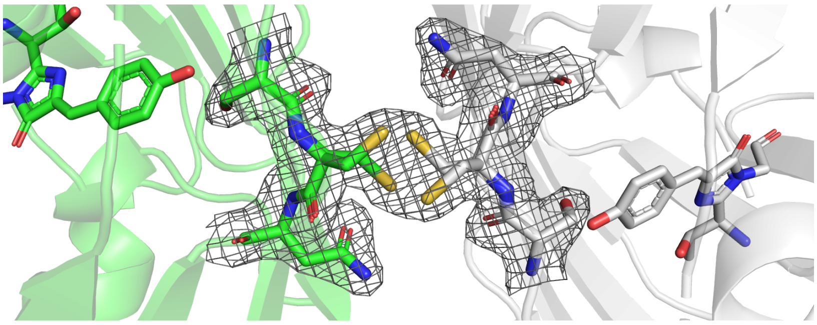

Fig. S8. The $2F_0$-F_c map (σ 1.0) of the disulphide bridge region connecting each subunit (green and grey) of dGFP-diS2.

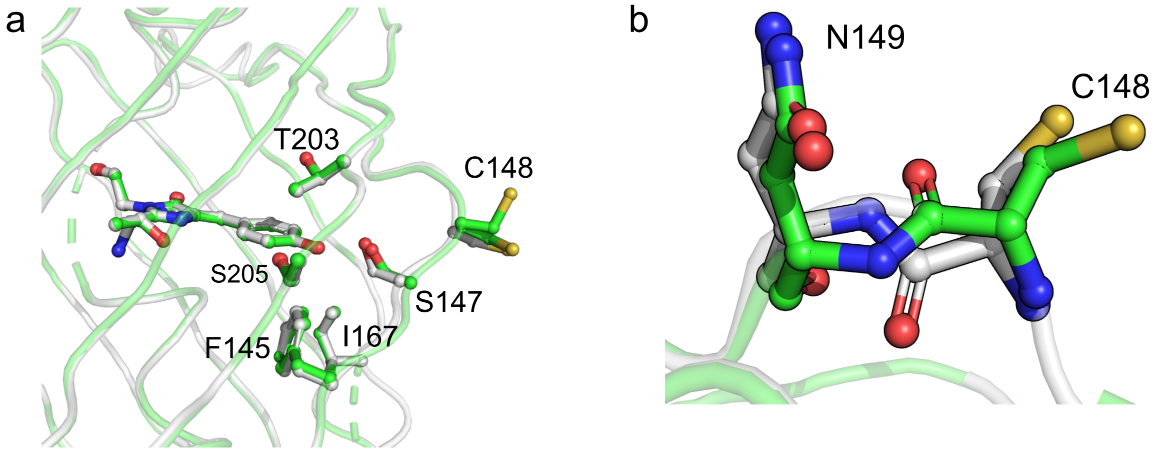

Fig. S9. Structural overlay of mGFP-diS2 (grey) and dGFP-diS2 (green) showing (a) key residues in close proximity to the chromophore and (b) C148-N149 peptide bond plane flipping.

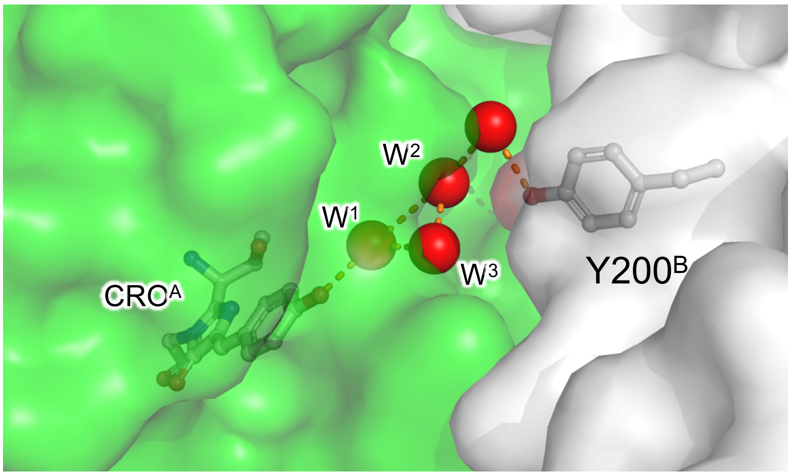

Fig. S10. Surface view of dGFP-diS2 showing buried waters at the water dimer interface. Subunits A and B are coloured green and white respectively. Waters are shown as red spheres and labelled as in Fig. 4 in the main text.

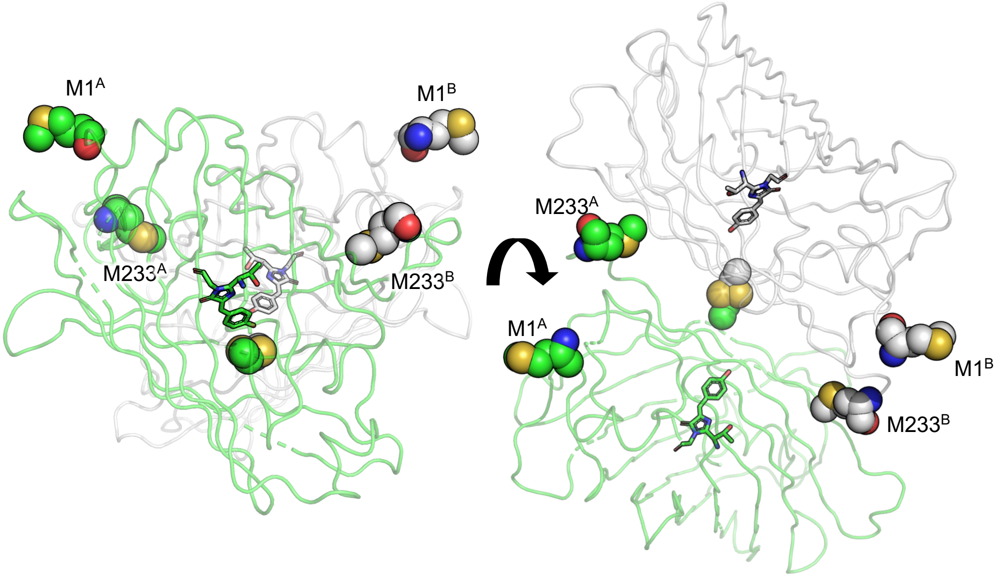

Fig. S11. Location of N-termini (M1^A and M1^B) and last observed C-terminal residue (M233^A and M233^B) in each monomer unit in dGFP-diS2.

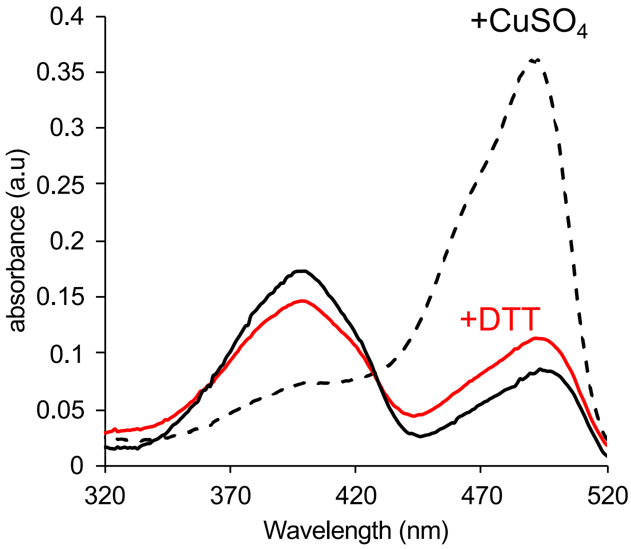

Fig. S12. The effect of DTT on GFP-diS2-ZIP. Absorbance spectra (5 μM protein) before (solid black line), after 60 min incubation with 1 mM $CuSO_4$ (dashed back line)and after 5 min incubation with 1 mM DTT (red line). After $CuSO_4$ addition, the GFP-diS2-ZIP was desalted prior to addition of DTT.

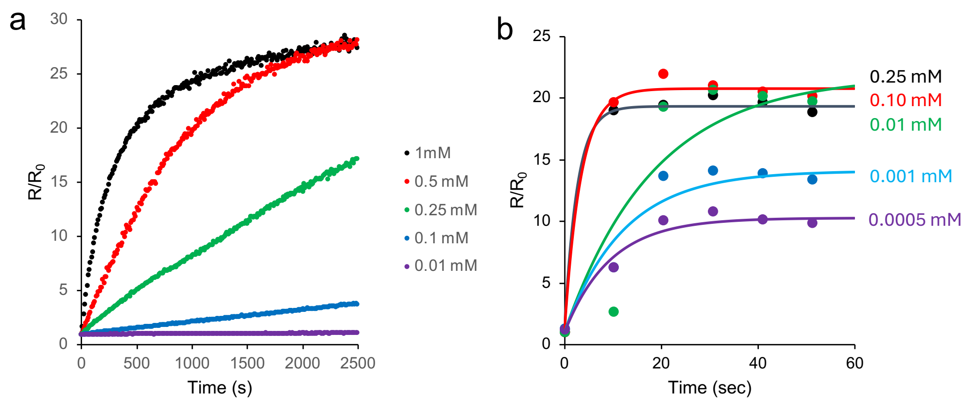

Fig. S13. Rate of change in fluorescence emission ratio ($Ex^{CRO\text{-}O^-}$:$Ex^{CRO\text{-}OH}$) in the presence of difference concentrations of (a) Cu^{2+} ($CuSO_4$) and (b) Cu^+ ($CuSO_4$ plus ascorbate). The copper concentration for each curve is shown on the plots themselves.

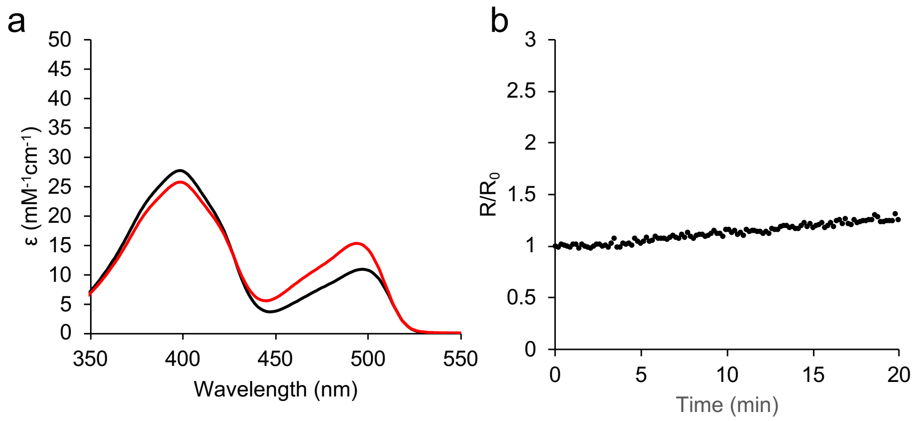

Fig. S14. The effect of ascorbate on the spectral properties of GFP-diS2-ZIP. (a) The absorbance spectra of GFP-diS2-ZIP (5 μM) before the addition of 500 μM ascorbate (black) and after 1 hr incubation (red). (b) The fluorescence emission ratio ($Ex^{CRO\text{-}O^-}$:$Ex^{CRO\text{-}OH}$) time course of 5 μM GFP-diS2-ZIP in the presence of 500 μM ascorbate.

Supporting References.

1 S. C. Reddington, E. M. Tippmann and D. D. Jones, *Chemical Communications*, 2012, **48**, 8419–8421.

2 E. Krissinel and K. Henrick, *J Mol Biol*, 2007, **372**, 774–97.

3 J. Wei, J. S. Gibbs, H. D. Hickman, S. S. Cush, J. R. Bennink and J. W. Yewdell, *Journal of Biological Chemistry*, 2015, **290**, 16431–16439.

4 H. S. Auhim, B. L. Grigorenko, T. K. Harris, O. E. Aksakal, I. V. Polyakov, C. Berry, G. dos P. Gomes, I. V. Alabugin, P. J. Rizkallah, A. V. Nemukhin and D. D. Jones, *Chem Sci*, 2021, **12**, 7735–7745.

5 H. L. Worthy, H. S. Auhim, W. D. Jamieson, J. R. Pope, A. Wall, R. Batchelor, R. L. Johnson, D. W. Watkins, P. Rizkallah, O. K. Castell and D. D. Jones, *Commun Chem*, 2019, **2**, 83.

6 J. D. Pédelacq, S. Cabantous, T. Tran, T. C. Terwilliger and G. S. Waldo, *Nat Biotechnol*, 2006, **24**, 79–88.
